# Nootkatone Supplementation Attenuates Carbon Tetrachloride Exposure-Induced Nephrotoxicity in Mice

**DOI:** 10.3390/antiox12020370

**Published:** 2023-02-03

**Authors:** Chongshan Dai, Mingchao Liu, Qinzhi Zhang, Subhajit Das Gupta, Shusheng Tang, Jianzhong Shen

**Affiliations:** 1 National Key Laboratory of Veterinary Public Health Security, College of Veterinary Medicine, China Agricultural University, Beijing 100193, China; 2 Beijing Key Laboratory of Detection Technology for Animal-Derived Food Safety, Beijing 100193, China; 3 College of Veterinary Medicine, Hebei Agricultural University, Baoding 071001, China; 4 Department of Internal Medicine, University of Texas Southwestern Medical Center, Dallas, TX 75230, USA

**Keywords:** nootkatone, oxidative stress, nephrotoxicity, Nrf2/HO-1 pathway, NF-κB pathway

## Abstract

Nootkatone (NKT), a major ingredient of Alpinia oxyphylla, exhibited potential nephroprotective effects; however, the precise molecular mechanisms remain poorly understood. This study aimed to study the nephroprotective effects of NKT and the underlying mechanisms in a mouse model. Our results showed that NKT pretreatment at the doses of 5, 10, and 20 mg/kg per day for 7 days significantly attenuates carbon tetrachloride (CCl_4_)-induced increases of serum BUN and CRE and kidney pathology injury. NKT pretreatment also markedly inhibited oxidative stress, inflammatory response, and the activation of caspases-9 and -3 in kidneys of mice exposed to CCl_4_. Meanwhile, NKT pretreatment downregulated the expression of NOX4, IL-1β, IL-6, and TNF-α proteins and NO levels in the kidney tissues. Moreover, NKT pretreatment upregulated the expression of Nrf2 and HO-1 mRNAs, and downregulated the expression of NF-κB, IL-1β, IL-6, TNF-α, and iNOS mRNAs in the kidneys of mice, compared to those in the CCl_4_ alone treatment group. In conclusion, our results reveal that NKT supplementation could protect against CCl_4_ exposure-induced oxidative stress and inflammatory response in the kidneys by inhibiting NOX4 and NF-κB pathways and activating the Nrf2/HO-1 pathway. Our current study highlights the therapeutic application of NKT for kidney diseases.

## 1. Introduction

The kidney is the most important excretory organ and plays a vital role in maintaining the stability of the internal environment in the body. Acute kidney injury is one type of kidney disease and it is a common clinical problem [1]. Usually, most of chemicals, chemotherapy drugs, or their metabolites could be eliminated via the urine after metabolism in the kidney [2]. Nephrotoxicity could be triggered by these toxic chemicals, drugs, or their metabolites, such as cisplatin, colistin, cadmium, copper, and aflatoxin B1 [2,3,4,5,6,7]. In addition, some toxic chemicals could produce potential nephrotoxic effects via the production of toxic metabolites in the liver [8]. Although acute kidney injury is a severe life-threatening condition, the effective treatment drugs are limited and result in a higher mortality in clinics [1,2]. Therefore, there is an urgent need for the development of effective drugs or new therapeutics to overcome life-threatening acute kidney injuries.

Carbon tetrachloride (CCl_4_) is one of the most common toxic substances that could induce acute liver or kidney injuries in rodents [9,10,11,12]. CCl_4_ exposure could easily and quickly induce acute liver or kidney injuries, which are usually considered as a classic model for the development of hepaprotective or nephroprotective agents in preclinical studies [12,13]. Previous studies have demonstrated that cytochrome 2E1 (CYP2E1), a metabolic enzyme that governs conversion of CCl_4_ to the highly reactive trichloromethyl (∙CCl_3_) and trichloromethyl peroxide radicals (CCl_3_O_2_⋅), was also expressed in tubular cells in the kidney [14,15]. Therefore, the production of deleterious reactive oxygen species (ROS), followed by its cascade reaction oxidative stress, and inflammatory response, are also the basis of CCl_4_ exposure-caused nephrotoxicity, which are similar with its hepatoxicity [14,15,16]. In addition, some studies also reported that several signaling pathways, including p53, mitogen-activated protein kinases (MAPKs), NF-E2-related factor (Nrf2), transforming growth factor-β1 (TGF-β1), and nuclear factor-kappa B (NF-κB), also contributed to regulate the acute kidney injury or renal fibrosis caused by CCl_4_ exposure [15,17,18], albeit the precise mechanisms remain unclear.

**Figure 1:**
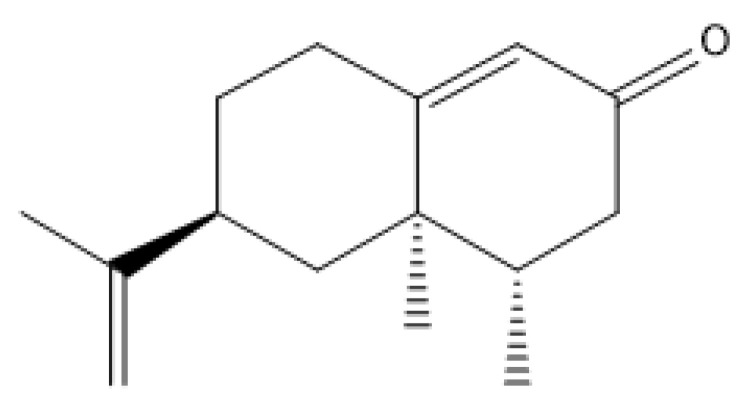
The image of nootkatone’s chemical structure.

Many studies have reported that medicinal plants could prevent nephrotoxic and hepatotoxic effects and they have become a vital resource for the development of new nephroprotective or hepaprotective agents [15]. Several investigations have reported that *Alpiniae Oxyphyllae* extract could effectively improve acute or chronic kidney injuries [19,20]. Nootkatone (NKT, the structure was shown in Figure 1) is the main active compound in the essential oil of *Alpiniae oxyphyllae* and has potent antioxidant and anti-inflammatory activities, which may partially explain the actional mechanisms of *Alpiniae Oxyphyllae* extract against nephrotoxicity or chronic kidney disease [21,22]. Previous studies showed that NKT exhibited potential neuroprotective, hepaprotective, and nephroprotective effects through inhibiting oxidative stress, blocking inflammatory response, and activating several signaling pathways for promoting cell survival, such as Nrf2 pathway, phosphoinositide 3-kinase (PI3K)/protein kinase B (PKB/Akt) pathway, and AMP-activated protein kinase (AMPK) pathway [22,23,24,25]. A recent study found that NKT supplementation at the dose of 10 mg/kg per day for 14 or 28 consecutive days could effectively inhibit the inflammatory response, apoptosis, and fibrosis in the kidney tissues in a unilateral ureteral obstructive mouse model by partly inhibiting the NAD(P)H oxidase-4 (NOX4)/ROS pathway and TGF-β1 pathway [21]. In another in vitro study, it was found that NKT supplementation at the final concentration of 50 μM could effectively activate the expression of heme oxygenase-1 (HO-1) protein, then inhibit lipopolysaccharide (LPS) exposure-induced the expression of inducible nitric oxide synthase (iNOS) protein, the production of nitric oxide (NO), and the release of high mobility group protein 1 (HMGB1) in Raw264.7 cells [26].

To date, there is limited data about whether NKT supplementation could improve acute kidney injury caused by exogenous toxic compounds. In this study, we explored the potential therapeutic effects of NKT supplementation on the acute nephrotoxicity caused by CCl_4_ exposure. The underlying molecular mechanisms focusing on oxidative stress, mitochondrial apoptotic, and NF-κB pathways were further studied.

## 2. Materials and Methods

### 2.1. Chemicals and Reagents

NKT (the purity is more than 97%) was purchased from Aladdin Reagent Company (Shanghai, China). Sodium carboxymethyl cellulose (CMC-Na) was obtained from Sigma-Aldrich Company (Shanghai, China). NKT was prepared a suspension with 0.5% CMC-Na) at the final concentrations of 2, 1, and 0.5 mg/mL for standby. CCl_4_ was obtained from Kaixing Chemical Company (Tianjin, China). The biochemical determination kits for catalase (CAT), superoxide dismutase (SOD), reduced glutathione (shown as GSH), glutathione peroxidase (GPX), iNOS, and malondialdehyde (MDA) were purchased from Nanjing Jiancheng Company (Nanjing, China). Mouse tumor necrosis factor-α (TNF-a), interleukin-1 beta (IL-1β), and IL-6 enzyme linked immunosorbent assay (ELISA) kits were purchased from R&D Systems Company (Minnesota, USA). NO Assay Kit and BCA™ Protein Assay Kit were provided by Beyotime Company (Haimen, China). The other reagents in this experiment were at least under the levels of analytical pure.

### 2.2. Animals and Treatments

All the animal studies in the current experiments were approved by the Institutional Animal Care and Use Committee from China Agricultural University and the approved number is CAU20220601-1. During experiments, mice were given adequate food and water and reared at a standard animal house that has a control temperature (at the range of 25 °C) and humidity (at the range of 55%) under a 12-h light/dark cycle.

**Figure 2:**
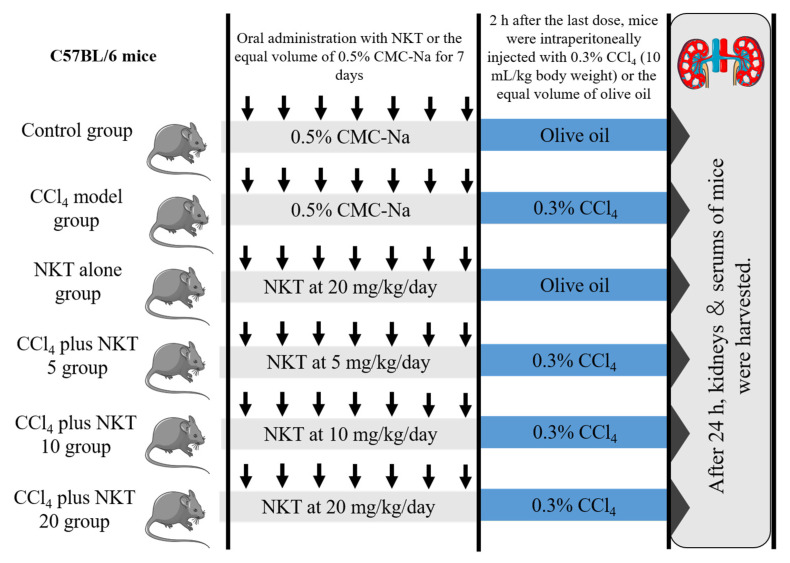
The schematic protocol for the experiment design.

Forty-eight male C57BL/6, weighted at 20–22 g (8-weeks-old), were randomly divided into six groups (eight mice in each group). A schematic protocol for the experiment design was shown in Figure 2. After 24 h after CCl_4_ injection, mice were sacrificed with pentobarbital sodium at the dose of 80 mg/kg body weight (intraperitoneal injection). Blood and kidney samples were obtained from the mice. The blood samples were used to analyze the measurement of biochemical parameters. One part of kidney samples was cut for the histopathological examination and the rest of the parts were stored in −80 °C for the expression analysis of proteins and genes.

### 2.3. Measurement of Blood Urea Nitrogen (BUN) and Creatinine (CRE) Levels

To assess the changes of mouse’s kidney function, the blood samples were collected using a 1.5 mL-size sterile Eppendorf tube. Then, the blood samples were centrifuged at 3000× *g* for 15 min. After centrifugation, the serum samples were isolated. An analyzer (Hitachi 7080, Hitachi Ltd., Tokyo, Japan) was employed to measure the levels of blood urea nitrogen (BUN), and creatinine (CRE), according to the descriptions in a previously published study [9].

### 2.4. Histopathological Assessment

The isolated renal tissues were fixed in 4% neutral formaldehyde for at least 48 h, then were treated according to the previous study for histopathological assessment. A semi-quantitative score (SQS) system was performed, according to the published descriptions [27].

### 2.5. Biochemical Analysis in the Kidney Tissues

Kidney tissues (about 50 mg) were homogenized with 0.5 mL of PBS in a 1.5 mL-size sterile Eppendorf tube using a High-Speed Low Temperature Tissue Grinding Machine (Sercivebio Company, Wuhan, China). After homogenate treatment, samples were centrifuged at 12,000× *g* for 15 min at 4 °C and the supernatants were scanned to measure the levels of MDA, GSH, and NO, and the activities of CAT, SOD, iNOS, and GPX, according to the kit manufacturer’s instructions. The protein concentration of each sample was determined using a BCA™ protein assay kit. The levels of MDA, GSH, and NO, and the activities of CAT, SOD, iNOS, and GPX in each sample were normalized to the corresponding protein concentration.

### 2.6. Measurement of the Biomarkers of Inflmatory Response

The biomarkers of inflammatory response, including the levels of IL-1β, TNF-α, IL-1β, and IL-6 proteins, were examined using the commercial IL-1 β, TNF-a, and IL-6 ELISA kits, respectively, according to our published study [10].

### 2.7. Measurement of the Activties of Caspases-3 and -9 Activities

About 50 mg of kidney tissues were lysed with 0.5 mL lysis buffer at −10 °C using a High-Speed Low Temperature Tissue Grinding Machine (Sercivebio Company, Wuhan, China). Then, samples were centrifuged to collect the supernatants (12,000× *g*, 15 min, 4 °C). The activities of caspases-3 and -9 were examined by using the commercial kits. The protein concentration of each sample was determined using a BCA™ protein assay kit. The levels of caspases-3 and -9 activities in each sample were normalized to the corresponding protein concentration.

### 2.8. Immunohistochemical Examination

Immunohistochemical examination was employed to measure the expression of NOX4 protein in the kidney tissues of mice, according to our previous study. The basal protocol was strictly followed according to previously published descriptions [28]. A rabbit polyclonal antibody against NOX4 antibody (1:200; ProteinTech, Chicago, IL, USA) and a goat anti-rabbit IgG (1:200; Santa Cruz, Dallas, TX, USA) were used. The staining results of NOX4 were assessed by using a semiquantitative scores, i.e., score 0 indicates none, score 1 indicates weakly positive staining with pale yellow or light brown color, score 2 indicates positive staining with the brown color, and score 3 indicates the strongly positive with dark brown or tan color. Twenty different areas of each slice were photographed and the average values of each score were calculated.

### 2.9. Quantitative Reverse-Transcription (qRT) PCR

A commercial total RNA Isolation Kit (No. RC112-01, Vazyme Biotech Co., Ltd., Nanjing, China) was employed to obtain the total RNAs and the protocol was followed the manufacturer’s instructions. A Nanodrop reader (Thermo Fisher Scientific, Shanghai, China) was used to assess the quality of RNAs. 1 μg RNA of each sample were employed to synthesize the cDNA by using an RT-PCR kit (Takara, China) the protocols were followed according to the manufacturer’s instructions. The primers of genes, including mouse NF-κB, IL-1β, TNF-α, IL-6, iNOS, NOX4, Nrf2, and HO-1, were provided by OriGene Company (Wuxi, China). Detailed information is presented in Supplementary Table S1. A real-time PCR instrument (AB7500, USA) was used to measure the expression of targeted genes. β-actin was the control gene. The 2^−ΔΔCt^ method was used to obtain the relative transcript abundance of these targeted genes.

### 2.10. Statistical Analysis

All data of this current study are reported as mean ± standard deviation (S.D.), unless otherwise specified. A statistical analysis was performed by using one-way analysis of variance (ANOVA) provided by GraphPad Prism 9.0 software (Graphpad Software Inc., La Jolla, CA, USA). Tukey’s multiple comparisons test was performed when the variance was homogeneous, otherwise, Dunnett’s T3 test was performed. Finally, a *p*-value less than 0.05 was considered as statistically significant.

## 3. Results

### 3.1. NKT Supplementation Attenuates CCl_4_-Induced Kidney Dysfunction of Mice

**Figure 3:**
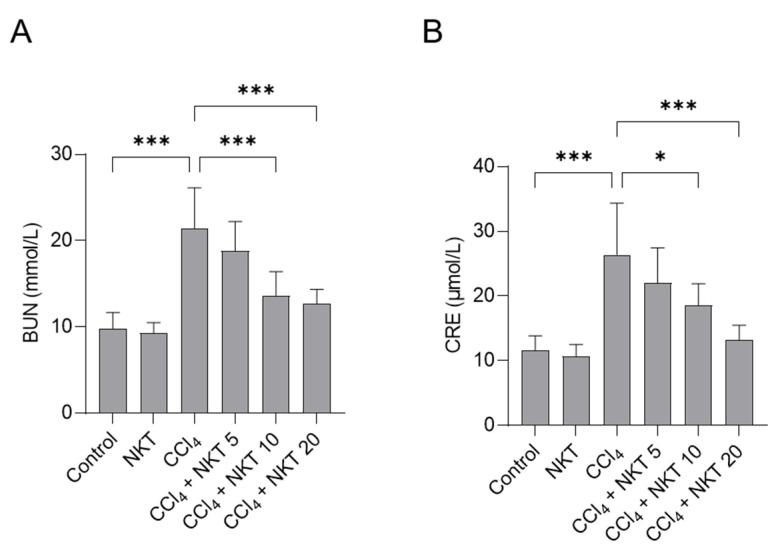
NKT pre-treatment improves CCl_4_ exposure -induced the increases of serum BUN (**A**) and CRE (**B**) levels. Data reported as mean  ±  S.D. (*n*  =  8 in each group). * *p*  <  0.05, and *** *p*  <  0.001.

To assess the kidney function, the levels of BUN and CRE in serum were determined. As shown in Figure 3, compared to the control group, CCl_4_ treatment markedly upregulated the levels of BUN and CRE to 21.4 mmol/L and 26.3 μmol/L (both *p* < 0.001), respectively. Compared to CCl_4_ model group, NKT pretreatment significantly improved the kidney dysfunction caused by CCl_4_ exposure. NKT pretreatment at 5, 10, and 20 mg/kg/day for a week markedly decreased the BUN levels to 18.8 mmol/L, 13.6 mmol/L (*p* < 0.001), and 12.7 mmol/L (*p* < 0.001), respectively, and reduced the levels of CRE to 21.9 μmol/L, 18.5 μmol/L (*p* < 0.05), and 13.2 μmol/L (*p* < 0.001), respectively. There was no marked change in the levels of BUN and CRE in the NKT alone treatment group, compared to those in the untreated control group.

### 3.2. NKT Treatment Attenuates CCl_4_ Exposure-Induced Pathology Damage in the Kidneys

**Figure 4:**
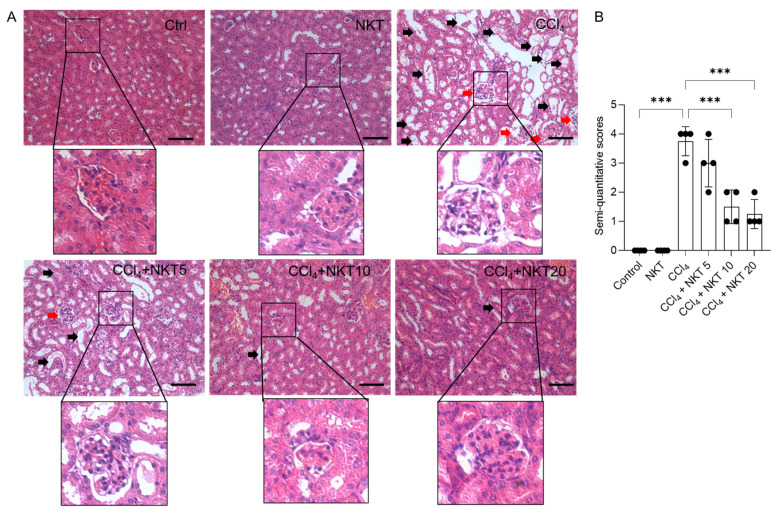
NKT pre-treatment improves CCl_4_ exposure-caused renal damage in mice. Mice were pretreated with NKT at 5, 10, and 20 mg/kg/day for seven successive days, then were treated with 0.3% CCl_4_. After 24 h, the histopathological changes were assessed. (**A**), the representative images were shown and a magnification of the glomerulus in each image were performed. (**B**), the result of semi-quantitative score. Data are presented as mean  ±  S.D. (*n* = 4 in each group). The black arrow indicated marked tubular degeneration, necrosis, tubular dilation, and cast formation. The red arrow indicated marked congestion and focal hemorrhage in glomerulus. *** *p*  <  0.001. Bar = 50 μm.

As shown in Figure 4, the marked histopathological changes were detected in the CCl_4_ model group, which were effectively attenuated by NKT supplementation. As shown in Figure 4, the marked pathology changes in the kidney tissues, including marked tubular degeneration, necrosis, tubular dilation, and cast formation, as well as marked congestion and focal hemorrhage of glomerulus and in the CCl_4_-treated mice, were detected. These pathology changes were markedly attenuated by NKT pretreatment, especially in the CCl_4_ plus NKT 10 and CCl_4_ plus NKT 20 groups. Correspondingly, in the CCl_4_ plus NKT 10 and CCl_4_ plus NKT 20 groups, markedly decreased SQSs were detected (from 3.75 to 1.50 and 1.25, respectively) (both *p* < 0.001), compared to the CCl_4_ model group. Compared to the control group, NKT treatment did not cause marked damage in the kidney tissues of mice.

### 3.3. NKT Treatment Attenuates Oxidative Stress Caused by CCl_4_ Exposure in the Kidneys

**Figure 5:**
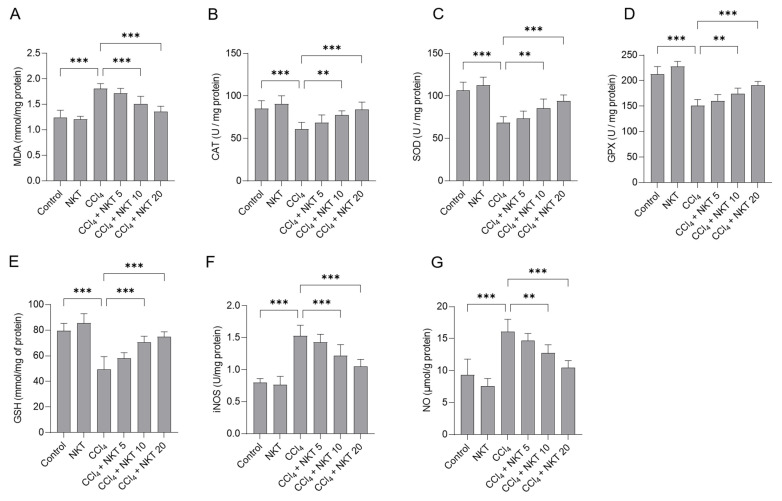
NKT pre-treatment attenuates CCl_4_ exposure-induced oxidative damage in the kidneys of mice. Mice were treated first with NKT at 5, 10, and 20 mg/kg/day for seven successive days, then were treated with 0.3% CCl_4_. After 24 h, MDA levels (**A**), and the activities of catalase (CAT) (**B**), superoxide dismutase (SOD) (**C**), glutathione (GSH) (**D**), glutathione peroxidase (GPX) (**E**), and inducible nitric oxide synthase (iNOS) (**F**) activities, and levels of nitric oxide (NO) (**G**) in the kidney tissues were measured, respectively. Data are shown as mean  ±  S.D. (*n*  =  8 in each group). ** *p*  <  0.01, and *** *p*  <  0.001.

We further measured the changes of oxidative stress biomarkers in the kidney tissues. As shown in Figure 5, compared to the control group, CCl_4_ exposure markedly increased the levels of MDA and NO and decreased the levels of GSH, and the activities of SOD, CAT, GPX, and iNOS in the kidney tissues of mice. In the CCl_4_ group, MDA and NO levels increased to 1.81 mmol/mg protein, and 16.1 μmol/g protein (both *p* < 0.001), compared to those in the untreated mice. Moreover, CCl_4_ treatment significantly decreased GSH levels to 49.5 mmol/mg of protein, and decreased CAT, SOD, and GPX activities to 60.9 U/mg protein, 68.8 U/mg protein, and 150.6 U/mg protein, respectively, and increased iNOS activities to 1.53 U/mg protein (all *p* < 0.001), compared to the control group. NKT supplementation effectively abolished oxidative stress damage in the kidney tissues of mice exposed with CCl_4_. Treatment in the range of 10 or 20 mg/kg/day a week starkly reduced the MDA levels to 1.51 mmol/mg protein and 1.35 mmol/mg protein (Figure 5A), respectively; significantly increased the CAT activities to 77.7 U/mg protein and 84.1 U/mg protein, respectively (Figure 5B); significantly increased the SOD activities to 85.5 U/mg protein and 94.1 U/mg protein (Figure 5C); significantly increased the GPX activities to 174.2 U/mg protein and 190.9 U/mg protein (Figure 5D); significantly increased the GSH levels to 70.5 mmol/mg of protein and 74.8 mmol/mg of protein (Figure 5E) (*p* < 0.01 or *p* < 0.001), respectively. Meanwhile, NKT addition at 10 and 20 mg/kg/day for one week also significantly reduced iNOS activity to 1.22 U/mg protein and 1.05 U/mg protein (Figure 5F), respectively; and significantly decreased the NO levels to 12.7 μmol/g protein and 10.5 μmol/g protein (Figure 5G), respectively, compared to the CCl_4_ model group. NKT treatment alone did not adversely affect the levels of MDA, NO, and GSH, and the activities of SOD, CAT, GPX, and iNOS in the kidney tissues of mice.

### 3.4. NKT Treatments Attenuates Inflammatory Reponse Induced by CCl_4_ Exposure in the Kidneys of Mice

**Figure 6:**
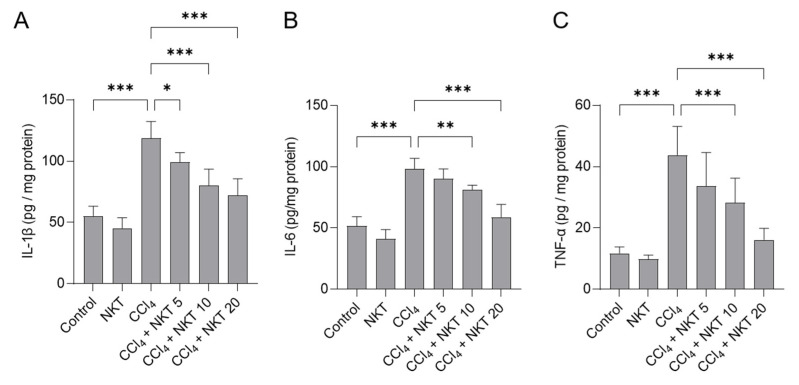
NKT pretreatment improves CCl_4_ exposure-induced inflammatory response in murine kidney tissues. Mice were pre-treated with NKT at 5, 10, and 20 mg/kg/day for seven successive days, then were treated with 0.3% CCl_4_. After 24 h, the levels of IL-1β (**A**), IL-6 (**B**), and TNF-α (**C**) proteins in these tissues were determined, respectively. Data are shown as mean  ±  S.D. (*n*  =  8 in each group). * *p*  <  0.05, ** *p*  <  0.01, and *** *p*  <  0.001.

Next, we measured IL-1β, IL-6 and TNF-α protein levels in the kidneys of mice. As shown in Figure 6, compared to the control, CCl_4_ exposure significantly increased the levels of IL-1β, IL-6 and TNF-α proteins of kidneys to 119 pg/mg protein, 98.1 pg/mg protein, and 43.8 pg/mg protein (all *p*  <  0.001), respectively. NKT supplementation provided the effectively inhibitory effects for the expression of these three inflammatory factors. In the CCl_4_ plus NKT 10 and CCl_4_ plus NKT 20 groups, the levels of IL-1β protein were decreased to 80.1 pg/mg protein and 72.1 pg/mg protein, respectively; the levels of IL-6 protein were reduced to 81.3 pg/mg protein and 58.9 pg/mg protein, respectively; the levels of IL-6 protein were decreased to 28.3 pg/mg protein and 16.1 pg/mg protein, respectively. NKT treatment at the dose of 20 mg/kg/day for 7 days did not affect the expression of any of these above-mentioned inflammatory factors, compared to the control group.

### 3.5. NKT Treatment Attenuates CCl_4_ Exposure-Caused Caspase Activation

**Figure 7:**
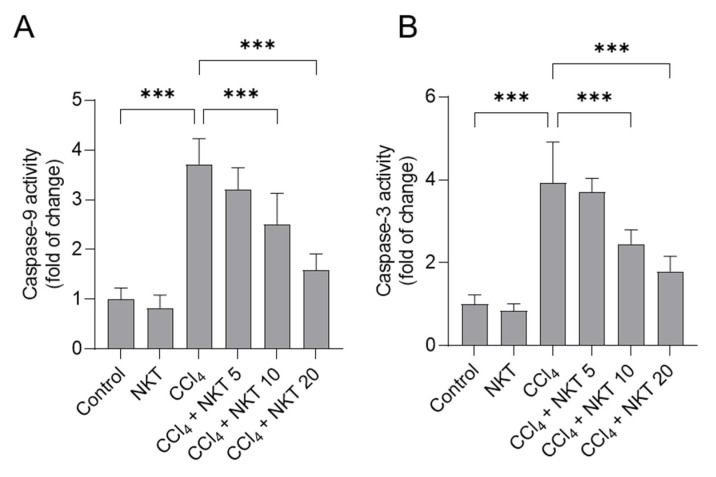
NKT pretreatment decreases the levels of caspases-9 and -3 in the kidneys of CCl_4_ -treated mice. Mice were treated as described in the previous figures. After 24 h, the levels of caspases-9 (**A**), and caspase-3 (**B**) in the kidney tissues of mice were determined, respectively. Data are shown as mean  ±  S.D. (*n*  =  8 in each group). *** *p*  <  0.001.

As shown in Figure 7, CCl_4_ alone treatment significantly increased caspase-9 and caspase-3 activities to 3.7-, and 3.9-fold, respectively, compared to those in untreated control mice. Compared to the CCl_4_ model group, NKT supplementation could effectively attenuate CCl_4_ exposure-induced the activation of caspases-9 and -3 in a dose-dependent manner. In the CCl_4_ plus NKT10 and CCl_4_ plus NKT20 groups, the levels of caspase-9 were decreased to 2.51- and 1.59-fold, respectively, and the levels of caspase-3 were decreased to 2.45- and 1.79-fold, respectively, compared to those in the CCl_4_ model group. NKT alone treatment at 20 mg/kg/day did not change the levels of caspases-9 and -3 in the kidneys of mice, compared to the control mice.

### 3.6. NKT Treatment Attenuates the Expression of NOX4 Protein in the Kidney Tissues

**Figure 8:**
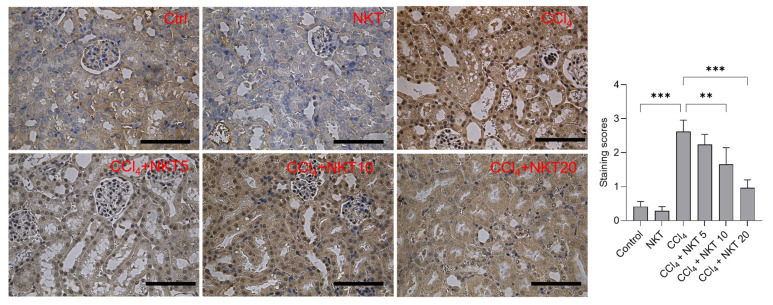
Immunohistochemical images of NOX4 protein in the kidney tissue. After treatment, the expression of NOX4 protein in the kidney tissues was measured by using an immunohistochemical staining method. The representative images (**on the left**) and semi-quantitative analysis (**on the right**) were shown. ** *p*  <  0.01 and *** *p*  <  0.001. Bar = 50 μm.

Compared to the control group, CCl_4_ treatment significantly increased the expression of NOX4, the staining scores increased to 2.62 (*p* < 0.001) (Figure 8). NKT pretreatment significantly dose-dependently decreased the expression of NOX4 protein. In the CCl_4_ plus NKT 10 and CCl_4_ plus NKT 20 groups, the staining scores significantly decreased to 1.66 (*p* < 0.01) and 1.97 (*p* < 0.001), respectively (Figure 8). In the NKT alone treatment group, NOX4 protein has a mild decreased expression, compared to the untreated control group (Figure 8).

### 3.7. NKT Treatment Downregulates the Expression of NF-κB, IL-1β, IL-6 TNF-α, iNOS and NOX4 mRNAs, and Upregulates the Expression of Nrf2 and HO-1 mRNAs

**Figure 9:**
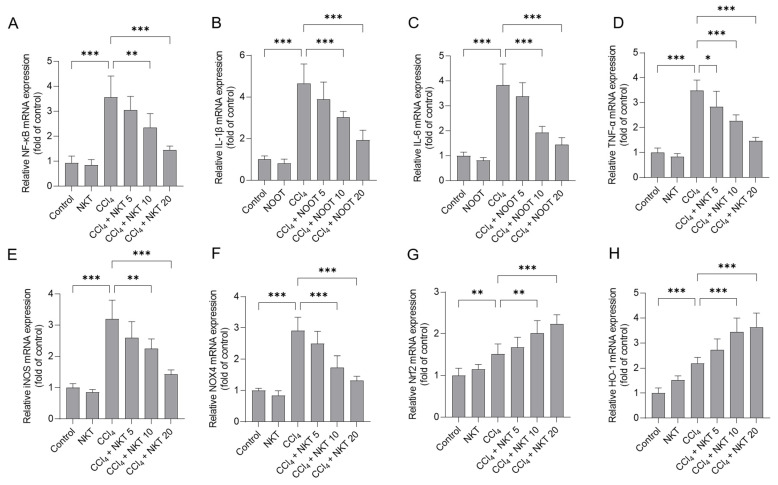
The effects of NKT pre-treatment on the mRNA expressions of different inflammation pathway genes in murine kidney tissues. After 24 h post CCl_4_ treatment, the expressions of NF-κB (**A**), IL-1β (**B**), IL-6 (**C**), TNF-α (**D**), iNOS (**E**), NOX4 (**F**), Nrf2 (**G**), and HO-1 (**H**) transcript levels in the kidney tissues were determined, respectively. Data are presented as mean  ±  S.D. (*n*  =  6 in each group). * *p*  <  0.05, ** *p*  <  0.01, and *** *p*  <  0.001.

CCl_4_ treatment significantly elevated the increased expression of NF-κB, IL-1β, IL-6 TNF-α, iNOS, NOX4, Nrf2, and HO-1 mRNAs in the kidney tissues of mice (Figure 9). NKT pre-treatment dose-dependently inhibited the expression of NF-κB, IL-1β, IL-6 TNF-α, iNOS, and NOX4 mRNAs, while activating the expression of Nrf2, and HO-1 mRNAs in the kidney tissues of mice. Especially, in the CCl_4_ plus NKT 20 group, the levels of NF-κB, IL-1β, IL-6 TNF-α, iNOS, and NOX4 mRNAs significantly decreased to 1.45-, 1.93-, 1.44-, 1.47-, 1.43-, and 1.32-fold, respectively, while significantly increased the expression of Nrf2 and HO-1 mRNAs to 2.23-, 3.64-fold (all *p* < 0.001), respectively, compared to the CCl_4_ model group (Figure 9). NKT alone treatment at 20 mg/kg/day mildly increased the expression of Nrf2 and HO-1 mRNAs, and mildly decreased the expression of NF-κB, IL-1β, IL-6 TNF-α, iNOS, and NOX4 mRNAs (Figure 9), compared to those in the untreated mice.

## 4. Discussion

Acute kidney injury, a part of acute kidney diseases and disorders, is defined by a sudden episode of the loss of excretory kidney function within short time, i.e., a few hours or a few days. An epidemiological investigation has shown that acute kidney injury-related mortality has increased at rates faster than that of breast cancer, heart failure or diabetes, causing a global public health stress burden [29]. It has been reported that acute kidney injury could be induced by environmental chemicals, drugs, or pathogenic microbes [7,29,30]. Acute kidney injury has been a global concern. Therefore, there is a necessary yet unmet medical need to develop the effective therapeutic drugs combating acute kidney injury.

CCl_4_ is a common environmental toxic compound and could cause marked liver and kidney dysfunction, in both humans, as well as rodent models [8,14,15,16,31,32,33]. In line with previous studies [8,31,32,33], in this current study, CCl_4_ exposure significantly increased the serum BUN and CRE levels, and induced marked pathology changes in renal tubules and glomeruli (Figure 3 and Figure 4), indicating acute kidney dysfunction. Moreover, our data (Figure 3, Figure 4, Figure 5, Figure 6, Figure 7, Figure 8 and Figure 9) further found that NKT supplementation could effectively improve acute kidney injury caused by CCl_4_ exposure, and the protective mechanisms involved a reduction in oxidative stress, NOX4, and NF-κB pathways with the concurrent accentuation of the Nrf2/HO-1 pathway.

Oxidative stress is an important basis of acute kidney injury caused by CCl_4_ exposure [8,31,32,33]. Previous studies showed that CCl_4_-induced oxidative stress in the kidney tissues of mice is partly dependent of the dichlorination metabolism based on CYP2E1 enzyme activities and the inhibitory effects on the endogenous antioxidant enzyme activities (e.g., SOD, CAT, and GPX) and antioxidants (e.g., GSH) [8,33]. CCl_4_ exposure could also induce marked lipid peroxidation, which was evidenced by the increased MDA levels in the kidney tissues [15,33,34,35]. The supplementation of natural products, including zingerone, naringenin, and taurine, could effectively inhibit lipid peroxidation and upregulated above-mentioned antioxidative enzyme activities or antioxidant levels in the kidney tissues, then improved renal dysfunction caused by CCl_4_ exposure [15,33,34,35]. Consistently, our current data presented that NKT supplementation at the final doses of 5 through 20 mg/kg per day for seven days could significantly inhibit the production of MDA, and upregulated the levels of GSH, and the activities of SOD, GPX, and CAT in the kidneys of mice (Figure 5). Similarly, a recent study from Chen et al. showed that oral NKT intervention at a dose of 10 mg/kg/day for 14 days or 28 days could significantly upregulate the activities of SOD and CAT in the kidneys, followed to protect against unilateral ureteral obstructive-induced renal damage in a mouse model. In vitro, NKT could stall the production of ROS, and enhance SOD and CAT activity, and finally inhibit hydrogen peroxide (H_2_O_2_)-induced oxidative stress and cytotoxicity in PC12 cells [36]. Taken together, these evidences indicate that NKT supplementation could improve renal oxidative damage via enhancing endogenous antioxidant enzymes and inhibiting lipid peroxidation. Additionally, several studies also showed that CCl_4_ exposure could upregulate the expression of NOX4 protein, which is a key protein of triggering intracellular ROS production, then inducing oxidative stress damage in the liver tissues [37,38]. NOX4 could be activated by transforming growth factor-β (TGF-β) or TNF-α, which are two pro-inflammatory factors in the process of acute liver or kidney injuries caused by CCl_4_ [39]. Our current study found that CCl_4_ exposure upregulated NOX4 mRNA and protein expression, which was dose-dependently downregulated by NKT supplementation (Figure 8 and Figure 9). A previous study from our group showed that pharmacological inhibition of NOX4 could effectively inhibit the mitochondrial ROS production, and subsequently protect against cell apoptosis caused by colistin [21]. It has been reported that NKT supplementation could inhibit the expression of NOX4 protein in unilateral ureteral obstructive-treated kidneys [21]. Therefore, the inhibition of NOX4 partly by NKT may contribute to explain its anti-oxidative effects caused by CCl_4_.

It is well known that apoptosis is a classic programmed cell death. Excessive ROS could not only cause oxidative stress, but also induce lipid, protein, and DNA damage [10,40,41,42]. In the current study, our results showed that CCl_4_ exposure significantly increased the activities of caspases-9 and -3 in the kidney tissues, which were partly blocked by NKT supplementation in a dose-dependent manner (Figure 7). Caspase-3 is an effector caspase and a biomarker of apoptosis [43]. Caspase-9 is a pivotal mediator of mitochondrial dysfunction-caused apoptosis (i.e., mitochondrial apoptotic pathway), which could directly cleave and activate caspase-3, to induce cell apoptosis [43]. Previous studies have illustrated that CCl_4_ exposure could result in marked mitochondrial dysfunction, in cascade to activating the expression of cytochrome C and release from mitochondria, then cleave and activate the caspase-9 through binding with apoptotic protease activating factor-1, finally triggering caspase-3-mediating cell apoptosis [9,10,39,44]. Very recently, it was found that NKT supplementation could effectively inhibit CCl_4_ exposure-induced the upregulation of caspase-9 and caspase-3 in murine liver [45]. Taken together, the data hinted that NKT supplementation could improve nephrotoxicity caused by CCl_4_ exposure by blocking caspase activation-mediated mitochondrial apoptotic pathway.

The anti-inflammatory activities of NKT have been illustrated in multiple studies [45,46,47]. The potential molecular mechanisms may be related to the downregulation of NF-κB, AMPK, and Toll-like receptor 4 pathways [22,46,48]. NF-κB is a key transcriptional factor, which could transcriptionally activate multiple pro-inflammatory cytokines, such as IL-1β, iNOS, TNF-α, IL-6, and cyclooxygenase-2 (COX-2) [49]. The upregulation of iNOS usually promoted the production of NO, which could react with superoxide (O_2_^•−^) to generate peroxynitrite, finally inducing an inflammatory response, or causing oxidative damage [50]. The elevated NO levels in the kidney tissues are an important indicator of the vascular endothelium injury, or the neutrophils activation [33]. For example, Nemmar et al.’s study showed that NKT pretreatment at a signal dose of 90 mg/kg could effectively inhibit the expression of NF-κB protein, then protect against diesel exhaust particles exposure-induced inflammatory response in the lungs of mice [51]. Xu et al. found that NKT administration via intraperitoneal injection at 10 mg/kg per two days for six weeks could effectively inhibit the activation of NF-κB, then inhibit the expression of IL-1β, IL-6, TNF-α, COX-2, and iNOS mRNAs, and the production of NO in the knee joint of mice [46]. Consistently, our current results showed that CCl_4_ exposure significantly upregulated the expression of NF-κB mRNA, and its downstream pro-inflammatory factors (e.g., IL-1β, IL-6, TNF-α, and iNOS) and the levels of NO in the kidney tissues. NKT supplementation significantly inhibited these pro-inflammatory factors and attenuated the congestion and focal hemorrhage in glomerulus (Figure 4, Figure 6 and Figure 9). It implied that the inhibition of NF-κB pathway partly contributed to the anti-inflammatory activities of NKT against nephrotoxicity by CCl_4_ exposure.

NKT may be a potential activator of Nrf2, which usually plays a protective role against oxidative damage in a context-dependent manner [22,48,52,53]. Under oxidative stress state, Nrf2 was released from the Nrf2-Keap1 complex in cytoplasm and transferred into the nucleus, wherein it transcriptionally upregulated the expression of genes such as SOD, CAT, GPX, HO-1, and NAD(P)H quinone oxidoreductase-1 (NQO1), offering a protective effect [54]. In this study, we see that CCl_4_ exposure significantly upregulated the mRNA expressions of Nrf2 and its downstream target HO-1, and NKT treatment further boosted their expression in the kidney tissues of mice (Figure 9). Previous studies have shown that Nrf2-gene-silencing or pharmacological inhibition of HO-1 could significantly sour the toxic effects caused by CCl_4_ exposure in liver [9,39]. Similarly, a recent in vitro study also found that the antioxidant and anti-inflammatory activities of NKT are partially dependent on the activation of the Nrf2 signaling pathway [22]. Meeran et al. found that NKT supplementation at 10 mg/kg/day for 10 days markedly upregulated the expression of Nrf2 and HO-1 proteins in the mouse’s heart, then effectively ameliorated the cardiotoxicity caused by isoproterenol exposure in a rat model [48]. In addition, many studies have illustrated HO-1 activation could inhibit the expression of NF-κB, via its end-products (i.e., bilirubin and CO), indicating the interaction between Nrf2 and NF-κB pathways participated in regulating the protective effect of NKT. This study provides the foundation for more research into deciphering the exact mechanisms, which would lead to effective therapeutic intervention in acute kidney injury.

## 5. Conclusions

**Figure 10:**
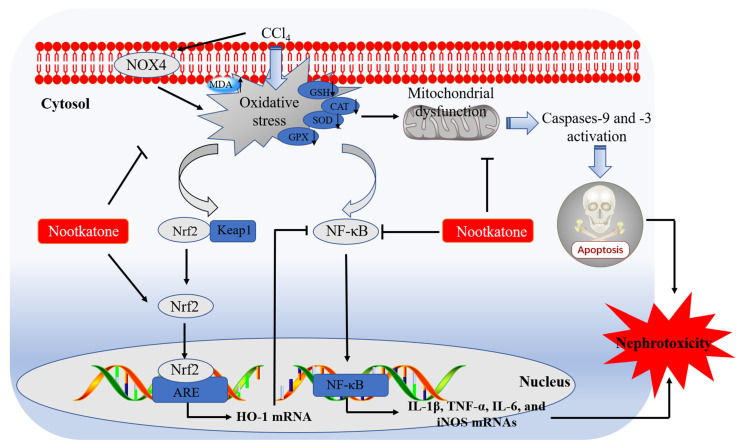
Pathways of NKT supplementation protecting against CCl_4_ exposure -induced nephrotoxicity. MDA, malondialdehyde; SOD, superoxide dismutase; CAT, catalase; GSH, glutathione; GPX, glutathione peroxidase; Keap1, kelch like ECH associated protein 1; Nrf2, NF-E2-related factor; HO-1, heme oxygenase-1; NOX4, NADPH oxidase 4; NF-κB, nuclear factor-kappaB; iNOS, inducible nitric oxide synthase; IL-1β, interleukin-1β; IL-6, interleukin-6; TNF-α, tumor necrosis factor-α; ARE, antioxidant response element.

In conclusion, our current data reveal that NKT could dose-dependently ameliorate nephrotoxicity caused by CCl_4_ exposure. It may involve the inhibition of oxidative stress and inflammatory response through blocking NOX4, mitochondrial apoptotic, and NF-κB, pathways, and promoting Nrf2/HO-1 pathways. A working model of NKT protecting against acute kidney dysfunction caused by CCl_4_ exposure is presented in Figure 10. Our current study provides new evidences and mechanistic insights for NKT as a potential candidate in treating kidney diseases. It also highlights that *Alpiniae oxyphyllae* is a beneficial Chinese herb or food in preventing or treating kidney diseases.

## Data Availability

The data are contained within this article.

## Supplementary Materials

The following supporting information can be downloaded at: https://www.mdpi.com/article/10.3390/antiox12020370/s1, Table S1: The primer sequences of targeted genes for qRT-PCR.
