# Supplementary material for: Nootkatone Supplementation Attenuates Carbon Tetrachloride Exposure-Induced Nephrotoxicity in Mice
Source: Antioxidants (Basel). 2023 Feb 3;12(2):370. doi: 10.3390/antiox12020370 (PMC9951873; doi:10.3390/antiox12020370)
Supplement: Supplementary file 1 [file antioxidants-12-00370-s001.zip › antioxidants-2194346-supplementary.pdf]

Supplymentary Table

Supplymentary Table S1. Primer sequences of the quantitative real-time PCR.

Gene name	Gene accession number	Direction	Primer sequence (5′to 3′)
IL-1β	NM_008361	Forward	5′- TGGACCTTCCAGGATGAGGACA -3′
		Reverse	5′- GTTCATCTCGGAGCCTGTAGTG -3′
IL-6	NM_031168	Forward	5′- TACCACTTCACAAGTCGGAGGC -3′
		Reverse	5′- CTGCAAGTGCATCATCGTTGTTC -3′
iNOS	NM_010927	Forward	5′- GAGACAGGGAAGTCTGAAGCAC -3′
		Reverse	5′- CCAGCAGTAGTTGCTCCTCTTC -3′
TNF-α	NM_001278601	Forward	5′- GGTGCCTATGTCTCAGCCTCTT -3′
		Reverse	5′- GCCATAGAACTGATGAGAGGGAG -3′
NOX4	NM_001285835	Forward	5′- CGGGATTTGCTACTGCCTCCAT -3′
		Reverse	5′- GTGACTCCTCAAATGGGCTTCC -3′
Nrf2	NM_010902	Forward	5′- CAGCATAGAGCAGGACATGGAG -3′
		Reverse	5′- GAACAGCGGTAGTATCAGCCAG -3′
HO-1	NM_010442	Forward	5′- CACTCTGGAGATGACACCTGAG -3′
		Reverse	5′- GTGTTCCTCTGTCAGCATCACC -3′
NF-kB	NM_008689	Forward	5′- GCTGCCAAAGAAGGACACGACA -3′
		Reverse	5′-GGCAGGCTATTGCTCATCACAG -3′
β-actin	NM_007393	Forward	5′- CATTGCTGACAGGATGCAGAAGG -3′
		Reverse	5′- TGCTGGAAGGTGGACAGTGAGG -3′
